# Optimizing the mnemonic similarity task for efficient, widespread use

**DOI:** 10.3389/fnbeh.2023.1080366

**Published:** 2023-01-26

**Authors:** Craig E. L. Stark, Jessica A. Noche, Jarrett R. Ebersberger, Lizabeth Mayer, Shauna M. Stark

**Affiliations:** ^1^Department of Neurobiology and Behavior, University of California Irvine, Irvine, CA, United States; ^2^Department of Cognitive Sciences, University of California Irvine, Irvine, CA, United States

**Keywords:** memory, pattern separation, hippocampus, aging, dementia, neuropsychological test

## Abstract

**Introduction:** The Mnemonic Similarity Task (MST) has become a popular test of memory and, in particular, of hippocampal function. It has been heavily used in research settings and is currently included as an alternate outcome measure on a number of clinical trials. However, as it typically requires ~15 min to administer and benefits substantially from an experienced test administrator to ensure the instructions are well-understood, its use in trials and in other settings is somewhat restricted. Several different variants of the MST are in common use that alter the task format (study-test vs. continuous) and the response prompt given to participants (old/similar/new vs. old/new).

**Methods:** In eight online experiments, we sought to address three main goals: (1) To determine whether a robust version of the task could be created that could be conducted in half the traditional time; (2) To determine whether the test format or response prompt choice significantly impacted the MST’s results; and (3) To determine how robust the MST is to repeat testing. In Experiments 1–7, participants received both the traditional and alternate forms of the MST to determine how well the alternate version captured the traditional task’s performance. In Experiment 8, participants were given the MST four times over approximately 4 weeks.

**Results:** In Experiments 1–7, we found that test format had no effect on the reliability of the MST, but that shifting to the two-choice response format significantly reduced its ability to reflect the traditional MST’s score. We also found that the full running time could be cut it half or less without appreciable reduction in reliability. We confirmed the efficacy of this reduced task in older adults as well. Here, and in Experiment 8, we found that while there often are no effects of repeat-testing, small effects are possible, but appear limited to the initial testing session.

**Discussion:** The optimized version of the task developed here (oMST) is freely available for web-based experiment delivery and provides an accurate estimate of the same memory ability as the classic MST in less than half the time.

## Introduction

We have known for many years that structures in the medial temporal lobe such as the hippocampus are not only critically involved in everyday memory for facts and events (Squire et al., 2004), but are key sites of age-related memory decline, Alzheimer’s disease (AD), and for potentially differentiating typical aging from AD even at its earliest stages (Small et al., 2011). Hippocampal-based memory in clinical settings is typically evaluated by trained neuropsychologists or neurologists using standardized standalone tests like the Rey Auditory Verbal Learning Test (RAVLT; Rey, 1941) or similar word-list learning measures in batteries like the CERAD (Morris et al., 1989). While performance on these types of tests certainly reflect memory impairments and functions of key structures like the hippocampus, they are not always sensitive to very mild impairments associated with prodromal stages of the disease. In addition, they can be significantly impacted by strategy use (which can increase variance and lead to practice effects), and often limited alternate forms exist, further complicating longitudinal testing.

**Figure 1:**
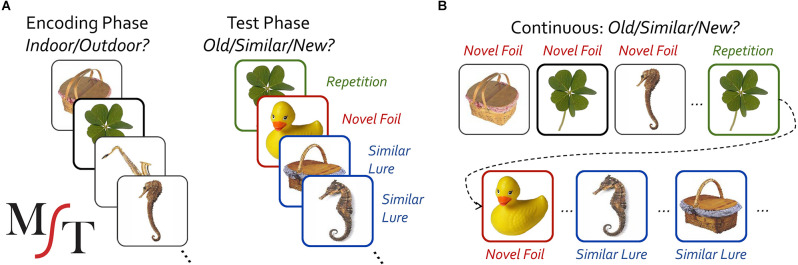
Schematic diagram of the study-test **(A)** and continuous **(B)** version of the Mnemonic Similarity *Task* (MST).

We have previously developed a modified recognition task, the **Mnemonic Similarity Task (MST)** (Kirwan and Stark, 2007; Stark et al., 2013, 2019), to assess hippocampal function using insights from a well-established, modern computational theory of the hippocampus’ use of pattern separation for rapid associative learning and memory (McClelland et al., 1995; Rolls and Kesner, 2006; Norman, 2010; Yassa and Stark, 2011). The MST modifies a traditional object-recognition memory task to include highly similar lure items that tax pattern separation and hippocampal function (Figure 1). The resulting measure, the “lure discrimination index” (LDI), has proven to be sensitive to hippocampal function, reliable, and highly tolerant of repeat testing.

Briefly, the LDI is impaired in amnesic patients with damage limited to the hippocampus, while recognition performance is intact (Kirwan et al., 2012). Likewise, hippocampal volume correlates with lure discrimination performance (Stark and Stark, 2017). In healthy volunteers, BOLD fMRI activity in the dentate gyrus (DG) and CA3 subfields of the hippocampus can discern the MST’s highly similar lure items from actual repetitions (Kirwan and Stark, 2007; Bakker et al., 2008; Lacy et al., 2010; Manelis et al., 2013)—a finding replicated in both spatial (Azab et al., 2014; Paleja et al., 2014; Reagh and Yassa, 2014) and emotional variants of the MST (Leal et al., 2014). Electrocorticography in humans has likewise shown hippocampal neurons discriminating between the lures and repetitions (Lohnas et al., 2018). Not only does the volume of the DG/CA3 correlate with lure discrimination over the course of development (Canada et al., 2019), but individual differences in DG/CA3 volume correlate with the impact of sleep deprivation on lure discrimination (Saletin et al., 2016) as well. Further, there is extensive evidence for age-related declines in lure discrimination on the MST (with recognition memory remaining intact; Toner et al., 2009; Holden et al., 2013; Stark et al., 2013, 2015; Doxey and Kirwan, 2015; Huffman and Stark, 2017; Stark and Stark, 2017), which correlates with age-related changes in DG/CA3 activity measured with fMRI (Yassa et al., 2010; Reagh et al., 2018). In addition, age-related changes in the integrity of hippocampal connectivity (e.g., fornix and perforant path) correlate with lure discrimination performance (Yassa et al., 2011; Bennett et al., 2015; Bennett and Stark, 2016) and diffusion metrics within hippocampal gray matter correlate with the LDI (Venkatesh et al., 2020; Radhakrishnan et al., 2022). Hippocampal hyperactivity has been observed using fMRI in individuals at greater risk for AD, including those with Mild Cognitive Impairment (MCI; Dickerson et al., 2004, 2005; Celone et al., 2006) and those carrying an APOE4 allele (Bookheimer et al., 2000; Trivedi et al., 2008; Dennis et al., 2010). Using the MST, individuals with MCI and AD have demonstrated deficits in lure discrimination performance beyond those simply associated with age (Yassa et al., 2010; Bakker et al., 2012, 2015; Ally et al., 2013; Stark et al., 2013), with additional impairments in traditional object recognition memory in MCI (Stark et al., 2013; Bennett et al., 2019) and AD (Ally et al., 2013). Likewise, impairments in lure discrimination have also been reported for carriers of the APOE4 allele (Sheppard et al., 2016; Tran et al., 2016; Sinha et al., 2018). Hyperactivity in DG/CA3 has also been linked to lure discrimination performance in MCI (Yassa et al., 2010; Tran et al., 2016; Sinha et al., 2018) and cerebral spinal fluid amyloid-β42 levels (Wesnes et al., 2014).

In interventions, a low dose of the antiepileptic drug levetiracetam, known to mitigate hippocampal hyperactivity in animal models (Koh et al., 2010), has been used in MCI patients, where it both showed a reduction in hippocampal hyperactivity (specifically the DG/CA3) and a concurrent improvement in lure discrimination performance on the MST (Bakker et al., 2012, 2015). Finally, recent work in the multi-site Anti-Amyloid Treatment in Asymptomatic Alzheimer’s Study, or the “A4 Study,” has shown that the Computerized Cognitive Composite or “C3” formed by tablet-based assessments including the MST, a one-card learning task (a variant of the MST using playing cards instead of objects), and a one-back task was the only reliable behavioral predictor found of Aβ− vs. Aβ+ status in cognitively normal adults (Papp et al., 2020). In repeat testing over several months, this same C3-composite was able to predict small (0.1 sd) changes in the Preclinical Alzheimer’s Cognitive Composite (PACC) measure of clinical decline (Jutten et al., 2022). Note, these studies not only use the MST to investigate aging and AD, but as a sensitive measure of hippocampal function to investigate a host of other disorders and conditions, including Schizophrenia, Major Depressive Disorder, radiation exposure, sleep deprivation, and pharmaceutical use (e.g., chemotherapy, drug use; Stark et al., 2019).

Thus, the MST has been widely adopted by the research community with over 100 articles and several clinical trials now using some variant of this task (Stark et al., 2019). However, the current design of the MST can be limited in its usefulness as a diagnostic tool for clinical applications. The current study-test design requires upwards of 15 min to administer and the use of specialized video instructions for accurate treatment of lure items. Here, our goal was to determine what strategies can be used to more efficiently estimate the LDI. In particular, we sought to determine the effect of the study-test vs. the more efficient continuous format, the effect of our traditional old/similar/new (OSN) vs. more easily understood old/new (ON) test instructions, and the effect of the distribution of trial types. Separately, we also sought to understand the effect of repeat testing, both when using unique stimuli in each test and when re-using stimuli across tests.

## Materials and methods

The MST has been extensively described elsewhere (Stark et al., 2013, 2019). Briefly, when used in the study-test format (Figure 1A), pictures of color objects appear on the screen (2.0 s, ≥ 0.5 s ISI) initially during an incidental encoding phase in which participants were asked to classify each object as belonging indoors or outdoors. For example, one might judge a picnic basket as an outdoor item and a rubber duck as an indoor item. The traditional full-length version uses 128 study items and is partially self-paced (the image disappears after the 2 s duration, but the prompts remain on screen until the participant makes a response with a minimum total trial length of 2.5 s). Immediately following the encoding phase, a test phase is given with an equal number of novel foils that are unrelated to any study items, true repetitions of study items, and similar lure items. Lure items have the same name as study items (names are not shown to the subject) but can vary in their similarity to the originally studied items and can be altered along a range of dimensions or be different exemplars (Stark et al., 2013, 2019). The continuous format version (Figure 1B) is similar, but uses a single phase, separating first and second (or lure) presentations out by typically 4–100 trials. In either format, participants’ memory can be probed with either a three-choice old/similar/new (OSN) response prompt (the ideal responses for repeat, lure, and foil trials respectively) or a two-choice old/new (ON) prompt (here, “new” would apply to both novel foils and similar lures). Both test structures and both choice formats have been used extensively in prior work (see Stark et al., 2019 for review). The MST has six independent sets of 192 image pairs with each pair having a particular degree of “mnemonic similarity”, derived from testing a large number of individuals and assessing the actual false alarm rate across individuals for image pairs and binning them into five lure-bin difficulty levels (Lacy et al., 2010).

The primary outcome measure of interest reflects a participant’s ability to discriminate similar lure items as being unique images rather than being a repetition of the studied item. In the OSN tasks, the measure is dubbed the LDI and equates to the probability of responding “similar” to the similar lure items minus the probability of responding “similar” to the novel foil items. This difference score helps to adjust for response biases. A parallel secondary outcome measure, dubbed REC, is a traditional “corrected recognition” score, equating to the probability of responding “old” to repeated items minus the probability of responding “old” to novel items. In the ON tasks, a signal detection theory framework is adopted (Stanislaw and Todorov, 1999). Here we create two different d’ measures to index discriminability, paralleling prior work (Stark et al., 2015). Paralleling the LDI, we compute d’(TL) to reflect how well participants can discriminate a true repetition from a similar lure (“old” responses to repetitions from the hit rate and “old” responses to lures from the false alarm rate). Paralleling the REC, we compute the d’(TF) to reflect how well participants can discriminate a true repetition from a novel foil (the same hit rate is used, but “old” responses to novel foils become the false alarm rate).

The traditional version of the MST has been freely available in several formats (stand-alone and online) on GitHub^1^. In prior work (Stark et al., 2021), we created an online version of the MST using the open-source jsPsych library for web-based deployment (de Leeuw, 2015) and the open-source JATOS package (Lange et al., 2015) to provide a reliable means of securely administering test sessions on the web and managing the data. We utilized the same structure here. All code is available at: https://github.com/StarkLabUCI/oMST-Data.

### Experiments 1–7

In each of Experiments 1–7, all participants received a full-length traditional MST (Study-test, OSN prompt, 128 study trials, 192 test trials, referred to as the “baseline MST”) and a modified version of the MST back-to-back in one session. Which test appeared first was counterbalanced across participants. For lure items, we used Set 1 and Set 2 from the MST, counterbalancing which was assigned to each test variant. Our primary outcome measure in these experiments was the correlation between the baseline MST’s LDI and the analogous measure in the modified version of the MST from the same subject. We used linear regression with automatic outlier detection (Prism 9.4.1, ROUT *Q* = 1%) to help address non-compliance with testing.

Prior to each phase came a set of instructions. Experiments 1–5 used the same video-based instructions used in many previous studies and made available on our lab website and on GitHub^1^. Experiments 6–7 shifted to a short series of guided practice trials in which participants are first told what to press for several stimuli before trying the task on their own for several more. Specifically, the study-test variant had four practice study trials and six practice test trials (three guided and three unguided) and the continuous version had nine practice trials (five guided and four unguided). Correct answers are forced on all trials and differences between studied items and lures are shown on all lure trials to clarify how participants should treat similar lure items.

Experiments 1–6 had participants recruited from UCI’s Human Subjects Lab Pool consisting of undergraduate students who participated for course credit. Participants were anonymous and were not screened. Experiment 7’s participants were older adults (mean *age* = 74 ± 8.3 years), recruited from several sources: an existing lab database, UCI’s Alzheimer’s Disease Research Center, and UCI’s Consent to Contact database. Cognitively normal, English-fluent older adults without prior history of neurological disorders or injury were included in the study.

Prior work in our lab with repeat testing on the standard MST has shown correlation coefficients ranging from 0.48–0.8 when comparing a full-length MST to variants shortened to 25–50% of the original length (Stark et al., 2015). Resolving a 0.48 correlation (*α* = 0.05, *β* = 0.2) requires a sample size of 32, which formed the minimum sample size used in each experiment. Participants were recruited in waves, however, and being done wholly online, data loss from poor engagement was anticipated. We recruited until, following analysis of a batch, at least 32 valid samples were present (see below), leading to sufficient, albeit unequal numbers of participants in each experiment.

Experiment 1 used two baseline MST tasks. Experiment 2’s variant was a continuous task with 256 trials using the OSN response prompt. A total of 128 of the trials were first presentations, 64 were similar lures, and 64 were repetitions. For both repetitions and lures, the gaps between the first and subsequent presentations included 32 trials with gaps of 4–11 and 32 trials with gaps of 20–99. Experiment 3’s variant was a full-length study-test, but shifted to the ON prompt. Experiment 4 combined both of these for a continuous ON test. Experiment 5a-b used the OSN prompt to test shortened versions of study-test (20 repetitions, 44 lures, and 20 novel foils) and continuous (64 1st presentations, 20 repetitions, and 44 lures) tasks. Experiment 6a-b replicated 5a-b but shifted away from our traditional video-based instructions to a guided practice task. Finally, Experiment 7 replicated 6b in healthy older adults.

We used the MST’s measure of traditional object recognition to filter participants who were not actively engaged in the study. In the OSN experiments, a minimum REC score of 0.5 was required. As this is a difference score (probability of responding “old” to repetitions minus the probability of responding “old” to novel foils), chance would be 0, but even older adults with Mild Cognitive Impairment typically score ~0.6 on this measure (Stark et al., 2013) making 0.5 a reasonable threshold for young, healthy adults. In the ON, the analogous metric is a d’(TF) score with a threshold of 1.5. Note, neither of these measures are our primary outcome measure.

### Experiment 8

In Experiment 8, each participant received four half-length traditional study-test sessions separated by approximately one week (minimum of 4 days). Here, we used Sets 1–5 with each set broken down into half-sized sets equating for difficulty (the traditional MST optionally uses the pre-determined difficulty to create matched-difficulty subsets). Half of the participants received unique stimulus sets in each session (e.g., 1a, 1b, 2a, 2b) while the other half received the same stimulus set every other session (e.g., 1a, 1a, 2a, 2a) to form our No-repeat and Repeat conditions.

Four-hundred and eighty three potential participants responded to a recruitment form from the online platform Reddit. These were initially screened *via* an email medical screening questionnaire to filter bots and participants with a history of brain injury or previous/current drug use. Subsequent screening *via* Zoom used the Montreal Cognitive Assessment (MoCA) to screen for general cognitive impairments. Seventy-eight adult volunteers were recruited after the screening process. After excluding six participants due to failure to complete all four sessions, the remaining 72 participants (28 males and 50 females; mean age = 31.4 ± 15.5 years) were divided into the Repeat (15 males and 24 females; mean age = 32.3 ± 14.3 years) and the No-Repeat group (13 males and 26 females; mean age = 29.5 ± 11.6 years). Over the course of 4 weeks, the participants were instructed to finish the MST online *via* a JATOS link. We again targeted at least 32 participants with valid data in each condition. Given the timing and long duration of the study, we recruited in batches and over-recruited both groups. A summary of Experiments 1–8 which includes the correlation coefficients for LDI from the traditional MST vs. the LDI from each alternate task variant is given in Table 1.

**Table 1:** For Experiment 1, subjects within each experiment performed two back-to-back sessions of the traditional, full-length MST that used the Study-Test format and Old, Similar, and New responses.

Exp	Goal	Alternate Task Variant	LDI Corr
1	Establish base test-retest reliability	Same task	0.73
2	Effect of continuous format	Full length, continuous, OSN	0.73
3	Effect of old/new response	Full length, study-test, ON	0.49
4	Combined continuous + old/new	Full-length, continuous, ON	0.56
5a	Effect of selectively reducing number of trials	Reduced, study-test, OSN	0.75
5b	Trials	Reduced, continuous, OSN	0.69
6a	Effect of shifting to guided practice instructions/trials	5a with practice task instructions	0.73
6b	Instructions/trials	5b with practice task instructions	0.73
7	Viability in older adults	6b’s reduced, continuous OSN	0.69
8	Multiple repeat testing	4x testing of study-test, OSN	N/A

For Experiments 2–7, subjects performed the traditional MST as well as a task variant that differed in task format (study-test vs. continuous), response prompt (OSN: Old/Similar/New vs. ON: Old/New), and length. Experiments 1–6 used young adults and 7 tested the resulting optimized version of the MST (oMST) in older adults. Experiment 8 compared repeat testing using the same set of stimuli vs. different sets of stimuli. LDI Corr = Pearson correlation coefficients (r) for correlations between LDI from the traditional MST and from each alternate task variant.

## Results

### Experiment 1: test-retest reliability of the baseline MST

**Figure 2:**
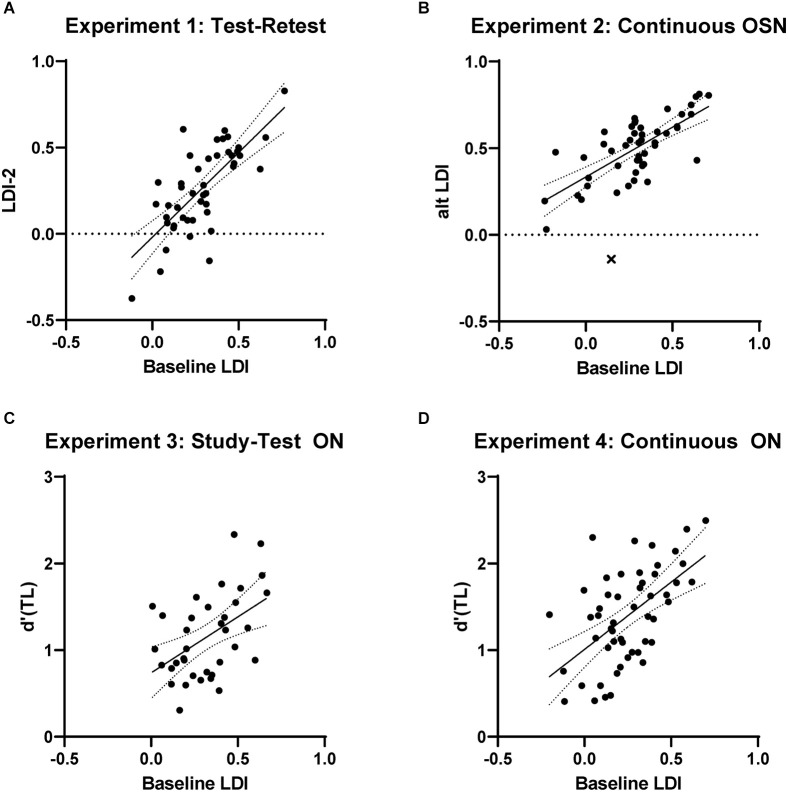
**(A)** Results from Experiment 1 showing the baseline test-retest reliability of the MST’s LDI. **(B–D)** Results from experiments 2–4 showing performance on the baseline, full-length, study-test MST’s LDI metric vs. alternate task LDI scores using: **(B)** the continuous format and old-similar-new prompt, **(C)** the study-test format and old-new prompt, and **(D)** the continuous format and old-new prompt. There is no apparent decrease in performance using the continuous task, but use of the old-new testing prompts reduces the correlation with baseline LDI. Points flagged as outliers indicated by “X”.

The goal of Experiment 1 was to determine the test-retest reliability of the traditional, full-length, study-test MST with its most-common OSN response prompt. A total of 60 participants enrolled in the study and 47 of these produced valid data (see Methods). The first MST task had an average LDI of 0.279 and the second had an average LDI of 0.271. A paired t-test showed this difference to not be reliable (*t*_(46)_ = 1.01, *p* = 0.29; Cohen’s *d* = 0.05). The critical correlation between these two however was quite strong, measuring 0.73 (Figure 2A, one outlier removed). This serves to establish a target for test-retest reliability of the MST under these testing conditions as the gold-standard test was used twice with unique stimuli (assessed using Fisher r-z tests vs. the value observed here).

### Experiment 2: effect of continuous test format

The goal of Experiment 2 was to determine the effect of shifting from the study-test format to the continuous format. The continuous format has two potential advantages. The first is that the experiment can be shortened considerably. A full-length study-test experiment has 320 trials in total and the continuous format only 256 (first presentations of later repeats and lures serve as the novel foils). Second, on each trial, participants must only ever make one decision and there is no need to refer to any prior study phase, potentially being somewhat clearer to participants and needing fewer instructions. A total of 65 participants enrolled in this experiment with 49 producing valid data.

This design also leads to a far shorter gap between initial exposure and subsequent test in the continuous version. With far fewer items and less time between, the continuous LDI here was 0.486 while the baseline LDI was 0.286. For our purposes here, however, this main effect is not problematic and can, in the case of testing impaired individuals rather than healthy young adults, be a benefit as it reduces floor effects. The critical correlation between the baseline MST and the continuous one was 0.73 (Figure 2B, one outlier removed), showing that shifting to a continuous format had no effect on reliability (Fisher one-tailed *p* = 0.99 vs. Experiment 1’s correlation).

### Experiment 3: effect of old-new response prompt

The goal of Experiment 3 was to determine the effect of shifting to the old-new instruction prompt. This has the potential advantage of being simpler to convey to participants as they do not need to understand what the experimenter means by a “similar” response in the traditional OSN response prompt. Participants are instructed to say “new” if there has been any change in the picture, no matter how minor they feel it might be. Here, we compared the baseline MST with an identical study-test version that only shifted the response instructions and choices. A total of 48 participants enrolled in this experiment with 36 producing valid data.

Here the baseline LDI measured 0.321 and the analogous d’(TL) from the ON test averaged 1.15. As these are different metrics, the baseline difference is not of interest, but the correlation between them is our critical measure. Here, the correlation dropped markedly from the prior experiments to 0.49 (Figure 2C), suggesting the change in test prompt and measure has the ON less reflective of baseline MST performance (Fisher one-tailed *p* < 0.05).

### Experiment 4: combining continuous format and old-new prompt

The goal of Experiment 4 was twofold. First, we sought to determine whether the drop in performance with the ON prompt was reliable, and second, we sought to determine whether there was any interaction with the test format. Here, we combined the manipulations from Experiments 2 and 3 to test a continuous, ON-based task vs. our baseline MST. A total of 61 participants enrolled in the experiment with 52 producing valid data.

The baseline LDI here averaged 0.247 and the continuous, ON d’(TL) averaged 1.392. The correlation remained lower at 0.56 (Figure 2D; Fisher one-tailed *p* = 0.08), suggesting that the shift to the ON prompt leads to a real drop in performance. As this correlation was no worse (and numerically better) than the study-test ON performance from Experiment 3, we can have more confidence that the continuous test format still results in a sensitive measure of discrimination for highly similar lures.

### Experiment 5: effect of selectively reducing the trial number

Experiments 1–4 have demonstrated that while the task format of study-test vs. continuous has no effect on how reliably it can assess the LDI, the response prompt clearly does. The ON prompt and d’(TL) measure do not accurately reflect baseline MST performance nearly as well. Optimization of the MST in Experiment 5 continued by focusing on using the OSN prompt and investigating whether the number of stimuli could be reduced to shorten the task without significantly impairing performance by focusing on maintaining a large number of the critical lure trials (20 repeats, 44 lures, and 20 foils).

**Figure 3:**
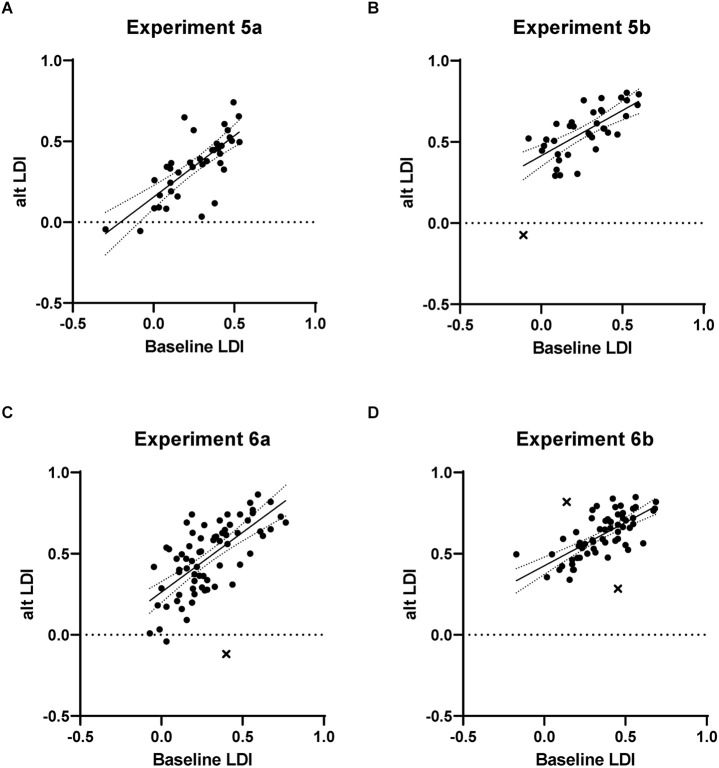
Results from Experiments 5 and 6 testing the effect of strategic reductions in the number of stimuli (Experiment 5) and the use of a practice task (Experiment 6). In both the study-test (panels **A** and **C**) and continuous (panels **B** and **D**) versions, strong correlations with the baseline MST were observed. Points identified as outliers are indicated with “X”.

Both study-test (Experiment 5a) and continuous (Experiment 5b) formats were tested. In Experiment 5a, 46 participants were enrolled, and valid data were available from 39. The average baseline LDI was 0.259 and the modified study-test task’s LDI was 0.351, with the slight increase likely resulting from the shortened delay between study and test items. The critical correlation between LDIs was high, at 0.75 (Figure 3A; Fisher one-tailed *p* = 0.84). In Experiment 5b, 49 participants were enrolled, and valid data were available from 37. The average baseline LDI was 0.262 and the modified continuous task’s LDI was 0.549, showing the anticipated overall increase consistent with the shorter lags in the continuous task. The critical correlation remained high at 0.69 (Figure 3B; one outlier removed; Fisher one-tailed *p* = 0.37). Thus, reducing the trial number still produced a reliable measure of discrimination in this task.

### Experiment 6: effect of guided practice trials

In Experiment 5, we reduced the number of stimuli overall but biased our sampling to over-represent the lures and were able to maintain a strong correlation with the baseline LDI scores. The video instructions we have used so far are very helpful in getting participants to understand the task and, in particular, what is meant by the “similar” response. However, the video is approximately 1.5 min and its instruction is passive. Here, to help ensure that participants understand the instructions, we shifted from using the video-based instructions to guided practice trials for each phase during the alternate tasks (the video instructions were still used in the baseline MST). In addition, as this represented a potential final version of the task, we sought to increase our sample size so that we might better understand any potential order or practice effects that occur given the fact that participants get two tests each. Finally, we sought to examine the time savings that result from both the reduced number of trials and from the altered instructional practice tasks. As in Experiment 5, for Experiment 6 we tested both the study-test (6a) and continuous (6b) formats.

In Experiment 6a, 85 participants enrolled, and valid data were available from 72. The average baseline LDI was 0.292 and the modified study-test task’s LDI was 0.469. The critical correlation between LDIs remained high, at 0.69 (Figure 3C; one outlier removed; Fisher one-tailed *p* = 0.36). In Experiment 6b, 82 participants enrolled, and valid data were available from 65. The average baseline LDI was 0.354 and the modified continuous task’s LDI was 0.612. The critical correlation remained high at 0.71 (Figure 3D; two outliers removed; Fisher one-tailed *p* = 0.43).

To examine the effectiveness of our attempts to reduce the total test time, we computed the median duration of each phase in both experiments (medians to reduce the effect of outlier points from pausing between task phases). In the baseline MST, the study phase instructions lasted 21.3 s and 24.3 s for 6a and 6b respectively and the study phases themselves lasted 240.7 s and 240.1 s for the total anticipated study duration of 4.39 min, collapsing across 6a and 6b. The baseline test phase instructions were 95.06 and 99.64 s with the phases themselves lasting 377.5 and 370.0 s, leading to an anticipated test duration of 7.85 min. The total duration for the baseline MST, therefore, was 12.24 min without breaks between components of the task. In contrast, using the revised instructions, the study instructions in 6a took a comparable 25.4 s while the test phase instructions took only half as long at 49.5 s. The study phase itself, with fewer trials, took 123.6 s and the test phase took 167.5 s, leading to a total of 6.1 min. This has cut the duration of the task in half. By shifting to the continuous format in 6b, the single instruction task took 64.7 s and the task itself took 251.0 s for a total of 5.26 min (43% of the baseline MST).

In Experiment 1, we were able to investigate the effect of order well given the within-subject design, and found only a trend towards worse performance on the second task. Here, our larger sample size afforded us a similar opportunity. In Experiment 6a, the baseline MST showed a small, but unreliable improvement when it was the second task (LDI = 0.277 vs. 0.310; *t*_(70)_ = 0.7, *p* = 0.47; Cohen’s *d* = 0.16). Similarly, the modified MST showed a small, but unreliable improvement when it was the second task (LDI = 0.449 vs. 0.492; *t*_(70)_ = 0.817, *p* = 0.42; Cohen’s *d* = 0.19). Experiment 6b showed a small, reliable increase in performance in the baseline MST when it was the second task (0.300 vs. 0.406; *t*_(63)_ = 2.6; *p* < 0.05; Cohen’s *d* = 0.65) and also showed a small increase in the modified continuous task’s LDI (0.576 vs. 0.649, *t*_(63)_ = 2.2, *p* < 0.05; Cohen’s *d* = 0.55). Thus, in a back-to-back testing format, there was some task learning that could be applied for a mild improvement in discrimination on the second administration in 6b, but not in 6a.

### Experiment 7: assessing the oMST’s viability in older adults

**Figure 4:**
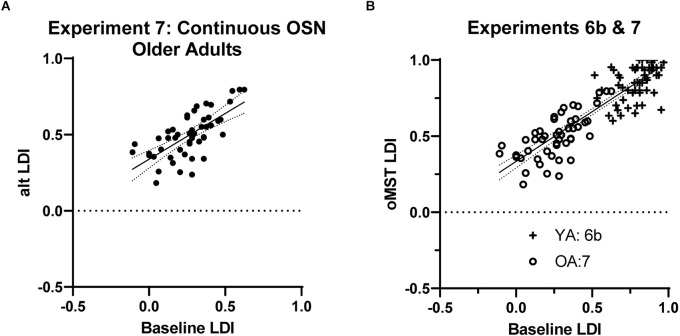
**(A)** Results from Experiment 7 in older adults showed similar reliability of the reduced, continuous, OSN task as shown previously. **(B)** In a direct comparison, combining data from this experiment and from the analogous Experiment 6b in younger adults, we find a single model fits the combined data well (*r* = 0.88).

In Experiment 7, we sought to perform initial testing of the viability of the shortened version from Experiments 5–6 in healthy older adults. Given its efficiency, we tested the continuous version of the shortened task from Experiment 6b. A total of 57 participants were enrolled and 50 produced valid data (two subjects were manually removed for extreme over or under-use of the “similar” response). The average baseline LDI was 0.265 and the modified continuous task’s LDI was 0.492. The critical correlation remained high at 0.69 (Figure 4A; Fisher one-tailed *p* = 0.36), demonstrating that the efficiency gains did not significantly compromise performance.

We further evaluated the performance of this version of the task by examining its ability to resolve the well-documented decline in LDI performance associated with age (Stark et al., 2019 for review). To do this, we compared young vs. older adult LDI performance on the baseline MST and in the shortened MST between Experiments 6b and 7. The baseline MST reliably resolved the difference in performance between age groups (*t*_(113)_ = 2.97, *p* < 0.01) with an effect size (Cohen’s d) of 0.56. The alternative, reduced version also resolved the difference between age groups (*t*_(113)_ = 4.49, *p* < 0.0001) with a somewhat larger effect size of 0.84. Thus, the reduced MST is still sensitive to age-related decline in lure discrimination.

Finally, we also compared performance here with performance in younger adults in Experiment 6b to determine whether the shift to this optimized format had a similar effect in younger and older adults. As shown in Figure 4B, younger and older adult performance on the two variations of the task can be well-captured by a single regression. Specifically, testing whether the data from the two experiments were better fit by separate linear regressions or by a common one using an extra sum of squares F-test suggested the single model be selected (*F*_(2,108)_ = 2.24, *p* = 0.11). That model suggests that using a y-intercept of 0.33 and a slope of 0.65 could be used to convert between baseline MST and oMST performance with reasonable accuracy (combined *r* = 0.88). We should note that within this model, while the correlation within the older group remained virtually unchanged (along with the slope and intercept), the correlation within the younger group data from Experiment 6b was reduced (intercept = 0.52, slope = 0.53, *r* = 0.43).

### Experiment 8: effect of multiple-repeat testing

Both previous research and the data from Experiments 1 and 6 provide a mixed view as to whether performance on the MST improves with repeated testing. The goal of Experiment 8 was to assess this impact directly in two ways and in a more representative population. Here, we recruited participants from internet forums into a study that tested their traditional, baseline MST performance once a week for four weeks. In addition to this basic repetition of the test experience itself, however, half of the participants were assigned to a condition in which the same exact test was given on weeks 1 and 2 and on weeks 3 and 4 (the “Repeat” group). The other half (the “No-repeat” group) received tests containing different stimuli each week.

The No-repeat condition ended with 34 participants with valid data and 108 valid sessions while the Repeat condition ended with 38 participants with valid data and 117 valid sessions. Data from both conditions were entered into a mixed effect model that could account for the missing sessions. The model had factors for test sessions (1–4) and for the group with separate models run for the LDI and REC measures.

**Figure 5:**
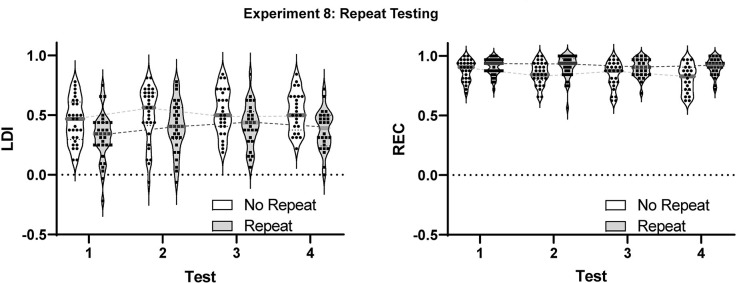
Results from Experiment 8 examining the effect of multiple testing on the LDI and REC measures in the MST. A modest, but reliable effect was observed in the LDI between the first test and all other test sessions. Violins show distribution with individual subject data included. Gray bars represent median values.

For the LDI (Figure 5), we observed both a main effect of test session (*F*_(2.696,132.1)_ = 8.294, *p* < 0.001) and of group (*F*_(1,70)_ = 9.190, *p* < 0.005) but no sign of interaction (*p* = 0.72). Thus, the No-repeat condition performed better than the Repeat condition consistently across all sessions. While somewhat unexpected, the presence of such an effect even in the first test and the distribution of scores showing a number of negative LDIs in the Repeat condition (Figure 5), suggests this main effect is the result of random assignment of particularly poorly performing participants to this group.

Critically, post-hoc multiple comparison testing (Tukey correction) revealed that, across groups, the first test session was reliably lower than each of the other three sessions (all adjusted *p*-values < 0.05), but that none of the other test sessions differed from each other (all adjusted *p*-values > 0.28). Thus, there was a small initial improvement following the first test, but performance remained constant thereafter. Interestingly, the same pattern was apparent in both groups. Thus, even when the identical stimuli were presented across weeks, performance remained constant and identical to the No Repeat condition. Had using the same stimuli itself led to a change in performance, test 4 should have differed from test 3 in the Repeat condition and the test 1 vs. test 2 difference in the Repeat condition should have differed from the test 1 vs. test 2 performance in the No Repeat condition.

For the REC measure (Figure 5), the main effects of the test session (*F*_(2.925,143.3)_ = 3.659, *p* < 0.05) and group (*F*_(1,70)_ = 8.549, *p* < 0.005) were again observed. However, in this case, the Repeat condition performed better than the No-repeat condition. Again, no interaction was present (*p* = 0.16). Post-hoc multiple comparison testing (Tukey correction) revealed only that the first session was mildly worse than the fourth (adjusted *p*-value < 0.05).

## Discussion

We set out to determine an efficient means of estimating the traditional, full-length MST’s LDI metric in a shorter task and, in the process, assess the effects of test format (study-test vs. continuous) and response prompt (old/similar/new vs. old/new). In remote, online testing, Experiment 1 established the test-retest reliability of the traditional MST’s LDI metric by testing individuals twice, back-to-back, using different stimuli. Experiments 2–7 kept this basic format but replaced one of the traditional MSTs with an alternate variant. While changing from study-test to a continuous format had no apparent effect on the reliability of capturing the LDI (Experiment 2), changing the response prompt to old/new markedly reduced the reliability (Experiments 3 and 4). As the LDI metric is based on responses to lures, we found that we could reduce the number of trials considerably from the traditional study-test (320 trials) to a reduced study-test (Experiment 5a: 148 trials) or continuous (Experiment 5b: 128 trials) with no effect on performance by focusing our reductions most heavily on the repeated and novel foil trials. Further efficiency was gained in both test formats by creating dedicated practice tasks *in lieu* of video instructions, again maintaining high reliability. In all, the combined running time of all instructions and task phases dropped by 57% by using this reduced, continuous format, which we now call the Optimized MST or oMST. We have made this version of the task available as web-based experiment on GitHub at: https://github.com/celstark/oMST.

Together, these results have demonstrated that while the old/new format does have its advantages in being easier to explain to participants, the task, or the d’(TL) measure used to capture how well participants can discriminate a similar lure from an actual repetition, raises some concern. While aging effects are readily apparent using this ON response prompt (e.g., Stark et al., 2015), the reduced ability of d’(TL) to reflect the LDI warrants caution in its use. From the data here, we cannot determine whether the reduced correlation with LDI is a bug or a feature, but other evidence suggests it is more the former. In particular, a re-analysis of the data from that article showed that Experiment 1’s OSN format yielded an effect size of 1.51 on the LDI’s ability to resolve an aging effect. Using similar recruitment and testing procedures, that article’s Experiment 4’s ON format yielded a lower effect size of 1.09 on the d’(TL) measure’s ability to resolve the aging effect. Thus, in the prior work (Stark et al., 2015), there is evidence that the ON format may be worse at resolving an aging effect. There are many reasons to refrain from comparing the age-related effect sizes observed in that article vs. the effect size extracted here. However, the lower effect size for the ON response prompt in Stark et al. (2015) is consistent with the ON prompt’s less reliable estimate of the LDI.

### Practice effects

In several prior interventional studies using young (Clemenson and Stark, 2015; Clemenson et al., 2019), middle-aged (Stark et al., 2021), and older adults (Clemenson and Stark, 2017; Kolarik et al., 2020; Wais et al., 2021), the MST’s LDI metric has shown no clear sign of practice effects. Yet, at least in the version used by CogState, at least two studies have shown not only practice effects (Papp et al., 2020) but the potential predictive power of such effects in diagnosing clinical cognitive decline (Jutten et al., 2022). It is worth noting that the version included in the C3 composite score used in these studies is a very short version (20 targets, lures, and repetition trials at test) and that the lures are tested in both their lure and their repetition format. But this alone cannot explain the differences and the results here do not present a clear understanding of when such effects are small but present and when they appear entirely absent. In Experiment 1, back-to-back testing in the same individuals shows an entirely unreliable numerical decrease of 0.008 in the second test. In using the shortened set with guided practice trials, results from the study/test version in Experiment 6a showed an unreliable increase of 0.033 when the MST came after an alternate version of the task, but results from the continuous version in Experiment 6b showed a reliable improvement of 0.106. Experiment 8, which sought to test this directly and in a more representative temporal lag of once per week, showed improvements of 0.069 and 0.090 in our two conditions between the first and second MST, but constant performance thereafter. Together, these suggest that there may be times in which there is an initial improvement in performance following the first time the test is administered. Perhaps learning how the test works and what the subjects are truly being asked to do when responding is the source of this. Experiment 8 did not have our new practice task and it is certainly possible that, combined with the remote testing, this induced the need for some participants to learn the task while performing it. This is, of course, somewhat speculative and why such “practice” or “testing” effects exist at some times and not others, is not entirely clear. But at least in our interventional work which failed to show such effects, testing was done in person, and it is certainly possible that the experimenter was able to instruct the participants more clearly as a result. Further research on this effect is certainly warranted.

However, one somewhat striking result from Experiment 8 is that repeating the test exactly from session to session when separated by a week had no effect whatsoever on the results. This lack of practice effects suggests that selecting stimuli from a unique set for each repeated test may not be necessary, and that experimenters or clinicians creating and administering repeated MST tests can imply randomly choosing appropriate distributions of trials and mnemonic similarity from across all six available sets.

### Deliverable

We set out to optimize the popular MST task to create a version that could capture its impactful LDI measure of hippocampal function more efficiently. The optimized oMST takes under half the duration and appears to be as effective as repeat testing of the baseline MST. As Figure 4B shows clearly, not only does this work well in older and younger adults, but the scores are readily convertible between versions using a simple regression fit. What’s more, the oMST has a benefit when testing older adults or impaired populations by reducing potential floor effects. Given the reduced lag between study and probe items, the overall performance is nicely raised.

The oMST is now freely available as a web-based task that can run on any computer or mobile device with a web browser using either keyboard presses or on-screen button clicks or touches for responses^2^. Unlike the traditional MST, it has a fully guided practice task to help ensure participants are clear on their instructions. We hope that, given the decreased task duration (now 5–6 min) and ease of use, the task will be more readily included in both basic and clinical research settings.

## Data availability statement

The datasets presented in this study can be found in online repositories. The names of the repository/repositories and accession number(s) can be found below: https://github.com/StarkLabUCI/oMST-Data.

## Ethics statement

The studies involving human participants were reviewed and approved by University of California, Institutional Review Board. The patients/participants provided their written informed consent to participate in this study.

## Author contributions

The initial design for the project was conceived by CS and SS and the choice of ongoing experiments was directed by CS. The manuscript was initially written by CS. All authors participated in data collection, quality control, analysis, and contributed to the final submitted manuscript and to its revisions. All authors contributed to the article and approved the submitted version.

## Footnotes

^1^ https://github.com/celstark/MST

^2^ https://github.com/celstark/oMST
